# Monkeypox mucosal lesions

**DOI:** 10.1016/j.idcr.2022.e01600

**Published:** 2022-08-17

**Authors:** Timothy Mathieson, Nicolas Dulguerov, Maxime Mermod

**Affiliations:** Hôpitaux Universitaires de Genève, Service d′Oto-rhino-laryngologie, 4 rue Gabrielle-Perret-Gentil, CH-1211 Genève, Switzerland

**Figure:**
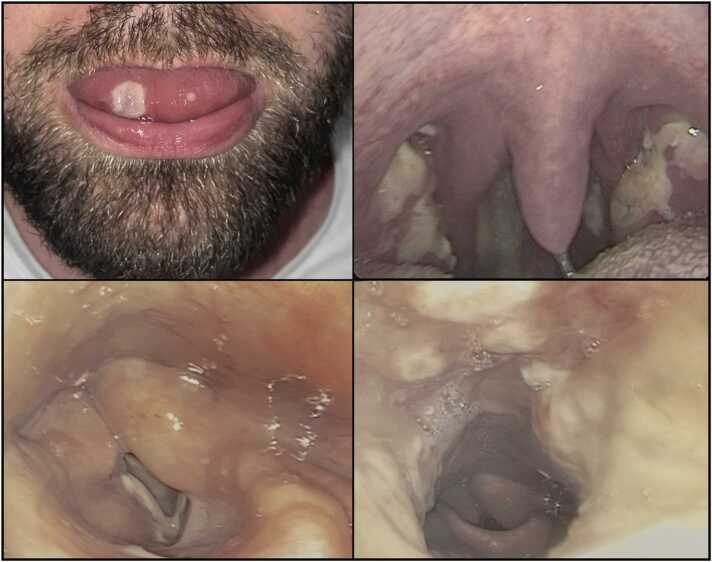

## Case illustrated

A 34-year-old man presented to the clinic with a five days history of sore throat. He had condomless oral intercourse with a new male partner three weeks earlier. On physical examination, necrotizing tonsillitis, pharyngitis and stomatitis with ulcerating vesicles on the anterior tongue (left upper panel), nasopharynx (right bottom panel), oropharynx (right upper panel) and laryngeal face of the epiglottis (left lower panel) were observed. Antiviral therapy for possible herpetic infection was initiated. Polymerase chain reaction assays of swabs obtained from pharynx lesions were positive for monkeypox and negative for herpes. Antiviral therapy for herpes was stopped, and the patient was treated by conservative measures. In classic cases of monkeypox, the first symptoms include fever, malaise, headache and sometimes sore throat and cough. In the current global outbreak of monkeypox, infections are being observed in persons who have had close contact with an infected person, particularly men who have sex with men. In the current case, mucosal lesions appeared seven days before cutaneous ones, suggesting close physical contact as the likely route of transmission during sexual contact. Within two weeks after his initial presentation, the patient’s lesions had abated without specific intervention.

## Ethics approval and consent to participate

Informed consent was obtained for publication of this case report and accompanying images.

## Funding

Publication made possible by support of the Department of Otolaryngology of Geneva University Hospital.

## CRediT authorship contribution statement

**Conceptualization**: Maxime Mermod and Timothy Mathieson, **Writing Original Draft preparation**: Maxime Mermod and Timothy Mathieson, **Supervision**: Nicolas Dulguerov, **Writing-Reviewing and Editing**: Maxime Mermod and Nicolas Dulguerov.

## Consent

Informed consent was obtained for publication of this case report and accompanying images.

## Declaration of Competing Interest

No conflicts of interest.

